# The association among 14-3-3η protein, inflammation, bone remodeling and osteoporosis in patients with rheumatoid arthritis

**DOI:** 10.12669/pjms.36.5.2403

**Published:** 2020

**Authors:** Yi Sun, Liang Hong, Changbai Gao

**Affiliations:** 1 Yi Sun, Department of Nephropathy and Rheumatology, Second Affiliated Hospital of Tianjin University of Chinese Medicine, Tianjin, 300250, China; 2 Liang Hong, Department of Surgery, Fourth Central Hospital of Tianjin, Tianjin, 300140, China; 3 Changbai Gao, Department of Nephropathy and Rheumatology, Second Affiliated Hospital of Tianjin University of Chinese Medicine, Tianjin, 300250, China

**Keywords:** 14-3-3η protein, Bone remodeling, Inflammation, Osteoporosis, Rheumatoid arthritis

## Abstract

**Objective:**

To evaluate the correlation among 14-3-3η protein, inflammation, bone remodeling and osteoporosis in patients with rheumatoid arthritis.

**Methods:**

In this cross-sectional study, the RA patients treated in our hospital were analyzed between January 2015 and November 2019. Bone mineral density was measured using dual energy X-ray absorptiometry, and at the beginning of the study, serum samples were collected and the level of 14-3-3η, TNF-α, and IL-6 was tested using the quantitative enzyme-linked immunosorbent assay, and I-CTX and PINP were measured using automatic electrochemical luminescence immune-analyzer for all the participants.

**Results:**

In the current study, 285 patients with rheumatoid arthritis were enrolled and assigned into normal, osteopenia, and osteoporotic group respectively. The level of 14-3-3η and IL-6 presented with the highest value in the osteoporosis group, but the lowest value in the normal group, and there were significant differences in the level of 14-3-3η and IL-6 among the groups (p<0.05), and there was positive correlation between 14-3-3η and IL-6 (p<0.05). There were significant differences in PINP and I-CTX among the three groups (p<0.05), and a significantly positive correlation between I-CTX and 14-3-3η (p<0.05) and a significantly negative correlation between PINP and 14-3-3η (p<0.05) were found.

**Conclusion:**

There was a significant correlation among 14-3-3η protein, inflammation, bone remodeling and osteoporosis in patients with rheumatoid arthritis, the influence of 14-3-3η on osteoporosis may be contributed to its adjusting inflammation and bone remodeling.

## INTRODUCTION

Rheumatoid arthritis (RA) is one of the most common chronic autoimmune rheumatic diseases which affects approximately 1.5% of the population in the world^1^, causes bone and joint damage including severe osteoporosis and disability, affecting the quality of life of patients adversely. Most researchers advocate that early diagnosis together with an accurate prognostic assessment, is crucial in the effective management of RA.^2^

14-3-3η proteins, a family of molecular chaperons, play an important role in the regulation of intracellular functions including proliferation, differentiation, metabolism, and many others.^3^ Among the proteins, the 14-3-3η protein has been regarded as a described and validated biomarker that has both a diagnostic and a prognostic value in RA.^1^ Moreover, in a study carried out by Gong, the level of serum 14-3-3η protein was found to be negatively correlated with bone mineral density (BMD) in RA patients, and serum 14-3-3η protein among groups of normal bone mass, osteopenia, and osteoporosis was significantly different, demonstrating 14-3-3η protein may affect the progression of osteoporosis in RA patients.^2^ Nevertheless, up to now, few studies have been focused on the mechanism by which 14-3-3η intervenes osteoporosis and the exact mechanism is still unclear.

Inflammation plays an important role in the development of osteoporosis. In a study performed by Al-Daghri and colleagues, 200 postmenopausal women aged 50 years and over including 100 with osteoporosis and 100 without were recruited, and serum IL-1β, IL-6 and N-telopeptides of collagen f-I (NTx) in women with osteoporosis were found to be significantly higher than the controls, IL-1β and TNF-α were positively associated with NTx in osteoporosis women, TNF-α and IL-6 were positively correlated with IL-lβ in both groups.^4^ In another study of 117 patients with RA, Sun and colleagues found Serum I-CTX and PINP were significantly correlated with BMD, TNF-α was significantly correlated with increased RANKL, and IL-6 was significantly correlated with low BMD in RA patients.^5^ These studies demonstrated a significant role of cytokine pattern-mediated inflammation in the occurrence and progress of osteoporosis. Based on these studies, we speculate that 14-3-3η protein may affect the level of inflammatory cytokines and the course of bone remodeling, to influence the occurrence and progression of osteoporosis. However, up to now, few studies have been performed on the issue in English literature.

Therefore, we conducted a cross-sectional study in our hospital. The aim of the study was to evaluate the correlation among 14-3-3η protein, inflammation, bone remodeling and osteoporosis, and determine the mechanism of 14-3-3η protein to affect the development of osteoporosis in patients with RA.

## METHODS

In this cross-sectional study, the RA patients treated in our hospital were analyzed between January 2015 and November 2019.

### The inclusion criteria

1. Patients were diagnosed with RA based on the 1987 revised criteria of the American College of rheumatology and the American College of Rheumatology/European league against Rheumatism EULAR/ACR 2010 criteria for RA.^2^
2. The patients agreed to participate the study and provided informed consents at the beginning of the study.

### Exclusion criteria

1. Patients with acute or chronic infectious disease such as HIV, liver or kidney disease, thyroid and parathyroid gland diseases.
2. Patients with other endocrinal disease.
3. Patients with concomitant use of androgens, steroids, anticonvulsant, estrogen, alcohol users, anticoagulant, or other causes which lead to secondary osteoporosis.^2^

The study was carried out in accordance with the Declaration of Helsinki, and approved by the ethics committee of our hospital at January 7, 2020 and an informed consent was provided by all the participants.

Bone mineral density (BMD) was measured using dual energy X-ray absorptiometry (DEXA) at lumbar spine L2-4 and proximal femur for all the participants.^2^ Based on the BMD T score, the patients were divided into three groups of normal, osteopenia, and osteoporotic groups according to world health organization (WHO) definition, i.e., BMD ≥ 2.5 standard deviations below the young adult mean (or T score ≤− 2.5) was defined as osteoporosis, and osteopenia was defined as BMD ≤− 1.0 SD and >− 2.5 SD.^2^

In addition, at the beginning of the study, serum samples were collected for all the participants in the morning after overnight fasting. Serum was separated immediately and stored. The level of 14-3-3η, TNF-α, and IL-6 was tested using the quantitative enzyme-linked immunosorbent assay (ELISA)^6^. The positivity of 14-3-3η was defined as ≥0.19 ng/ml according to the manufacturer’s recommended cutoff.^3^ I-CTX and PINP were measured using automatic electrochemical luminescence immune-analyzer^7^.

### Statistical analysis

Statistical analysis was performed using SPSS 22.0 (SPSS Inc., Chicago, IL, USA). The comparison of measurement data including 14-3-3η, TNF-α, IL-6, I-CTX and PINP was conducted via analysis of variance, and enumeration data was evaluated via Chi square test among groups. Multivariate logistic regression analysis was used to evaluate the associations among the variables. P < 0.05 was regarded as statistical significance.

## RESULTS

In the current study, 285 patients with RA who met the inclusion criteria were enrolled, in which 128, 80, and 77 patients were assigned into normal, osteopenia, and osteoporotic group respectively, based on the BMD values. There was no significant difference in gender distribution among the three groups (p>0.05), but as to age and disease course, significant differences were detected, and the osteoporotic group presented with a significantly elder age and longer disease course (p<0.05). The values of variables including age, gender, disease course, 14-3-3η, TNF-α, IL-6, I-CTX and PINP of the patients in the three groups show in Table-I.

**Table-I:** The characteristics and variables in three groups.

Variables	Normal group	Osteopenia group	Osteoporosis group	p-values
Number	128	80	77	-
Age (Year)	54.2±13.6	58.9±14.8	62.5±16.7	P<0.05
Gender (Male/Female)	49/79	29/51	28/49	p>0.05
Disease course (month)	32.6±11.8	65.5±27.6	92.3±36.7	P<0.05
14-3-3η (ng/ml)	0.28±0.09	0.45±0.11	0.91±0.21	P<0.05
TNF-α (pg /mL)	14.07±7.54	15.78±9.49	17.24±10.32	P>0.05
IL-6 (pg /mL)	7.5±4.2	8.4±4.9	10.6±5.2	P<0.05
I-CTX (ng/ml)	0.35±0.11	0.48±0.13	0.61±0.17	P<0.05
PINP (ng/ml)	49.5±10.8	39.9±8.7	28.5±5.9	P<0.05

**Fig.1:**
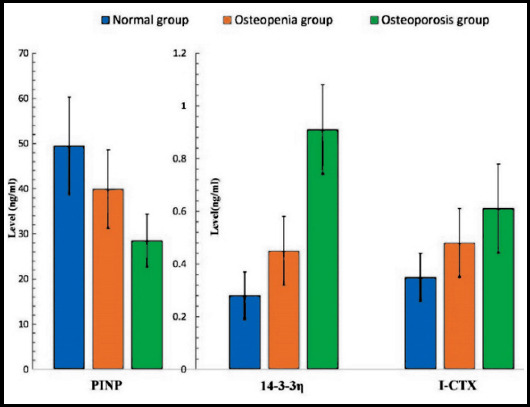
The distribution of PINP, 14-3-3η, and I-CTX in three groups.

**Fig.2:**
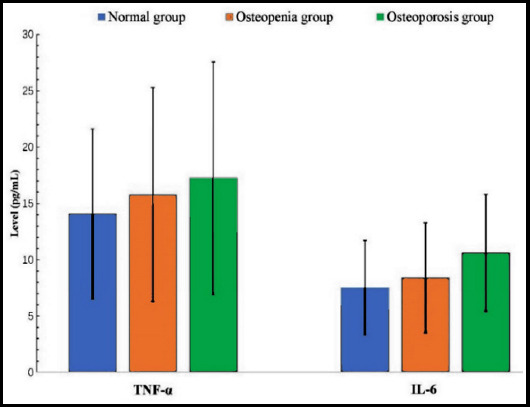
The distribution of TNF-α and IL-6 in three groups.

In the enrolled patients, 251 patients were positive in 14-3-3η and the positive rate was 88.1%. The level of 14-3-3η presented with a highest value in the osteoporosis group, but a lowest value in the normal group, and there was a significant difference in the level of 14-3-3η among the groups (p<0.05, Table-I). As to IL-6, its distribution showed the similar trend as 14-3-3η (p<0.05, Table-I, Fig.2). The mean of TNF-α also increased from normal group to osteoporosis group (Fig.2). However, different from IL-6 and 14-3-3η, there was no significant difference in the level of TNF-α among the three groups (p>0.05, Table-I, Fig. 1 and 2). In addition, among the patients there was positive correlation between 14-3-3η and IL-6 (p<0.05).

As to I-CTX, the value increased from normal group to osteoporosis group. Different from I-CTX, the value of PINP decreased gradually from normal group to osteoporosis group. There were significant differences in PINP and I-CTX among the normal, osteopenia and osteoporosis group (p<0.05, Table-I, Fig.2). Meanwhile, we found a significantly positive correlation between I-CTX and 14-3-3η (p<0.05), and a significantly negative correlation between PINP and 14-3-3η (p<0.05).

## DISCUSSION

In this study, we tried to evaluate the relationships among 14-3-3η protein, TNF-α, and IL-6, I-CTX and PINP, and analyzed the mechanism of 14-3-3η protein affecting the development of osteoporosis in patients with RA. To the best of our knowledge, few studies have been conducted in this issue in English literature.

Accurate and early diagnosis of RA and treatment as early as possible are of increasingly importance. In this study, the positive rate of 14-3-3η in the enrolled patients was 88.1%. However, the most commonly used laboratory indicators for RA, RF and anti-CCP antibody, the positive rate is reported to be about 70–80 and 70.0%^2^, demonstrating 14-3-3η presents with a higher sensitivity than RF and anti-CCP antibody. In Gong’s study, the sensitivity of 14-3-3η for RA was 91.7%^2^, and in Maksymowych`s study, the value was 77%.^8^ In this study, we didn’t enroll controls and evaluate the specificity of 14-3-3η, but as to sensitivity, our study was consistent with those previously published studies and further confirmed their outcomes.^2^,^8^

In a study carried out by Maksymowych, 14-3-3η titres from 33 patients with early RA and from 40 patients with established RA were measured, and the relationship between 14-3-3η level with radiographic damage and radiographic progression was investigated , and the results revealed that 14-3-3η expression was higher in the serum of patients with radiographic evidence of damage and progression.^9^ Osteoporosis is one of the signs of bone and joints damage in patients with RA.^2^ In Gong’s study, linear correlation analysis found that serum 14-3-3η protein was negatively correlated with BMD in RA.^2^ In the current study, we found there was significant difference in the level of 14-3-3η among normal, osteopenia, and osteoporosis group, and the normal group presented with the lowest level, the osteoporotic group presented with the highest level. Moreover, the correlation analysis also demonstrated 14-3-3η was significantly associated with BMD. Based on the current results, we found from normal to osteoporotic group, the level of 14-3-3η increased, which indicate that the three studies revealed the same outcome, i.e., 14-3-3η can affect the development of osteoporosis in RA patients.

Meanwhile, we found significant differences in the level of PINP and I-CTX among the normal, osteopenia and osteoporosis group as well as a positive correlation between I-CTX and 14-3-3η and a negative correlation between PINP and 14-3-3η. Both I-CTX and PINP are key biomarkers of bone remodeling.^10^ In addition, many studies have focused on the association between IL-6 and BMD, and advocated IL-6 play an important role in affecting normal bone metabolism and leading to osteoporosis.^11^ In Sun’s study, the level of IL-6 was found to be negatively correlated to BMD, and indicating chronic inflammation may increase the serum RANKLE, leading to the occurrence of osteoporosis.^5^ In the current study, we found there was significant difference in the level of IL-6 among the three groups, and in the enrolled patients there were positive correlation between 14-3-3η and IL-6. Subsequently, we concluded that the influence of 14-3-3η on the progression of osteoporosis may be contributed to its adjusting inflammation and bone remodeling.

### Limitations of the study

We excluded the patients with concomitant use of androgens, steroids, estrogen and some other drugs which may lead to osteoporosis, but in clinical practices many patients with RA may use these drugs, if these patients were enrolled, the current outcomes may be influenced.

### Recommendation

In this study some results were in line with previously published literature, but some were not. Hence, more studies should be carried out in the future to further resolve these issues.

## CONCLUSION

We concluded that 14-3-3η may affect the occurrence and progression of osteoporosis in RA patients via adjusting inflammation and bone remodeling course, but the details were still not clear.

### Author`s Contribution

**YS** conceived, designed and did statistical analysis,

**YS, LH, and CBG** did data collection and manuscript writing,

**YS and LH** did review and final approval of manuscript.
